# We need to talk: a qualitative inquiry into pathways to care for young men at ultra-high risk for psychosis

**DOI:** 10.3389/fpsyg.2024.1282432

**Published:** 2024-02-12

**Authors:** Håkon Olav Åmlid, Jan Carlsson, Jone Bjørnestad, Inge Joa, Wenche ten Velden Hegelstad

**Affiliations:** ^1^TIPS – Centre for Clinical Research in Psychosis, Stavanger University Hospital, Stavanger, Norway; ^2^School of Law, Psychology and Social Work, Örebro University, Örebro, Sweden; ^3^Department of Social Studies, Faculty of Social Sciences, University of Stavanger, Stavanger, Norway; ^4^Department of Psychiatry, District General Hospital of Førde, Førde, Norway; ^5^Institute of Public Health, Faculty of Health Sciences, University of Stavanger, Stavanger, Norway

**Keywords:** pathways to care, ultra-high risk, psychosis, men, help-seeking

## Abstract

**Introduction:**

It is known from the literature that men are slower to seek help and staying engaged in mental health care compared to women. Seeing that in psychosis, men more often than women have insidious onsets but also a more malign illness course, it is important to find ways to improve timely help-seeking. The aim of this study was to explore barriers and facilitators for help-seeking in young male persons struggling with early signs of psychosis.

**Methods:**

Qualitative interviews with nine young men who suffer from a first episode of psychosis or psychosis risk symptoms.

**Results:**

Male stereotypical ideals, significant others, and knowledge of symptoms and where to get help as well characteristics of symptom trajectories appeared to be important determinants of help-seeking behavior.

**Discussion:**

Interviews indicated that help-seeking in the participants was delayed first, because of reluctancy to disclose distress and second, because significant others were unable to accurately recognize symptoms. Information, awareness, and easy access to care remain important in early detection and intervention in psychosis and psychosis risk. However, more emphasis should be placed on de-stigmatizing mental health problems in men and aiming information specifically at them.

## Introduction

### Onset, course and outcome in ultra-high risk for psychosis

Modern research conceptualizes psychosis with a staging model. In this model, general mental health problems such as anxiety and depression are seen as early, while psychotic episodes are seen as later stages presenting with an admixture of signs and symptoms (van Os et al., 2010). A current goal of research is to enable intervention during the early stages, with the notion that this will prevent adverse outcomes. The Ultra-High-Risk (UHR) for psychosis state refers to a period of sub-clinical psychotic symptoms (Yung and McGorry, 1996; Insel, 2010; McHugh et al., 2018). It is associated with significant suffering and decline in cognitive, behavioral, and social function (McGorry et al., 2008). Being diagnosed with UHR is associated with a ~22% likelihood overall of transitioning into first-episode psychosis (FEP) (Fusar-Poli et al., 2012). This represents a substantially increased risk compared to the general population where the lifetime prevalence of psychosis is about 3% (Perala et al., 2007). Still, only a minority of patients present to mental health services before the stage of frank psychosis (Fusar-Poli et al., 2017; Ajnakina et al., 2019; Joa et al., 2021). According to recent research by Joa et al. (2021) and a recent meta-analysis by Fusar-Poli et al. (2020), only a meager 5–12% of at-risk individuals are detected before onset of a first episode of psychosis (FEP). Current strategies for identifying UHR thus appear inadequate.

More than half of individuals at UHR are men (58%) in adolescence or their early twenties, identified as having attenuated psychotic symptoms (APS) (Fusar-Poli et al., 2020). These symptoms typically include perceptual abnormalities and overvalued ideas. Help-seeking is often contingent on dramatic expressions such as suicidal ideation, self-harm, or suicide attempts (Judge et al., 2008; Rietdijk et al., 2011; Falkenberg et al., 2015). However, men more often than women, present with a long duration of untreated symptoms and insidious onsets characterized by negative symptoms such as blunted affect, reduced expression of emotion and social withdrawal (Rietschel et al., 2017). These are “quiet” symptoms that can be difficult to recognize. They are nevertheless important, as they may be markers of transition to psychosis and poorer outcomes (Bjornestad et al., 2022b).

### Health psychology and sociology of mental illness in men

Socially prescribed norms for traditional male gender roles can delay help-seeking (Galdas et al., 2005). In a qualitative study by Ferrari et al. (2018), findings indicated that gender stereotypes influenced the pathways to care for men and women differently. Women commonly sought help themselves, but experienced delays due to mistrust and misconceptions of the severity of their symptoms by the health care system. Men, on the other hand, commonly disclosed that they were stopped from seeking help because of male stereotypes such as perceived obligations to family, and a need for being “strong” and “in control.” Traditional male gender norms have typically rewarded anger, toughness, hostility, and emotional control. Consequently, vulnerable emotions such as sadness and fear have triggered shame, deterring help-seeking. Seeking help for mental health problems would for some men be associated with a perception of weakness and loss of control. Men are also sometimes concerned that disclosure of mental health problems could lead to social judgment or loss or bullying. This could be exacerbated if they had experienced negative responses to previous disclosure (Macdonald et al., 2022).

The notion that men are less likely than women to seek help for mental health issues has been contested by findings indicating that men do seek help but disengage from health services earlier than women (Seidler et al., 2016, 2021, 2022). In depression, Seidler et al. (2016) found that traditional masculine gender norms affect symptom types and expressions, attitudes, intentions, and behaviors related to help-seeking as well as what treatments and coping strategies men will commit to. Men will seek help and stay with it if it is accessible, engaging, and appropriate. Data from an online survey on experiences with therapy (*n* = 1907) found an overall drop-out rate of 44.8% (*n* = 855), whereof 26.6% (*n* = 120) dropped out after their first session. The most common reason for dropping out was a lack of connection with the therapist (54.9%), followed by the feeling of a lack of progress (20.2%). Younger age, unemployment, self-reported identification with traditional masculinity, and whether therapy gave participants a sense of emasculation, all predicted drop-out.

### Policy and organization of mental health care for men

Negative expectations of and beliefs about therapy form a barrier to help-seeking. Negative experiences with psychotherapy may cement male gender norms, deterring them from help-seeking. Inversely, past positive experiences with psychotherapy may mitigate male gender norms that act as barriers to help-seeking (Seidler et al., 2020). In one study investigating preferences for psychotherapy among men in a Canadian population (*n* = 92), 45.7% indicated a strong preference for the therapists to help them develop coping strategies, explore feelings and patterns of experiences, as well as assist in working with emotions. This finding opposes a commonly held belief that men are skeptical to therapies focusing on vulnerable emotions (Kealy et al., 2021). Prompting help-seeking in men who may need it requires information about what treatment may entail. One study indicated that some men may struggle to find information on available resources and services and which resources are of good quality (Macdonald et al., 2022). In a literature review on men’s health literacy, the authors found that approaches tailored to men’s everyday contexts and language were important for increasing the effectiveness of male health promotion programs. Information and treatment should address positive aspects of masculinity, as opposed to labeling masculinity as toxic (Rice et al., 2021). Researchers have emphasized the importance of further research and of including a male perspective when evaluating mental health services (Seidler et al., 2021). Providing treatment and information that men find engaging and satisfying may improve their confidence in the helpfulness of psychotherapy, consequently making them more likely to reach out for help (Seidler et al., 2020).

Despite knowledge about gender stereotypes and the way these may influence help-seeking behavior, men continue to be underrepresented in presenting to mental health services during psychosis risk (Wilson et al., 2017). More insight into mechanisms influencing help-seeking behavior in young men at psychosis risk, and subsequently how to encourage it, is warranted to accurately target and intervene in this group before the development of psychosis (Insel, 2010; Fusar-Poli et al., 2020). The aim of this study was to explore facilitators and obstacles of help-seeking in young men with UHR or FEP, using in-depth qualitative interviews.

## Materials and methods

A qualitative study into pathways to care employing open interviews with young men having entered mental health care for psychosis or psychosis risk. For the analytical process, we employed thematic analysis as described in Braun and Clarke (2013). Considering the exploratory nature of the study, as well as the limited body of research on the subject, we chose inductive thematic analysis, as we did not intend to fit the data within any prior coding frame nor the researchers’ analytic preconceptions.

### Setting

The study took place in Rogaland County of southwestern Norway, a catchment area encompassing ~300,000 inhabitants fulfilling age criteria for inclusion. It was catchment area based within the context of the Norwegian public healthcare system, providing free healthcare to all inhabitants regardless of income.

### Sample and recruitment

Recruitment was carried out by a detection team focusing on early detection of-and intervention in-psychosis and ultra-high risk through the TIPS Centre for Clinical Research in Psychosis. Participants were recruited from either The early detection and Intervention in Psychosis (TIPS-2) and Prevention Of Psychosis (POP) clinical research projects targeting the first-episode psychosis-and UHR populations, respectively. The projects employ early detection strategies in the form of an early detection team and information campaigns to reach the target groups. Nine men aged 16–25 were interviewed for the study. Criterion sampling was used, and participants were included if they identified as men, and had either been classified as UHR or FEP. All participants gave informed consent, and the project was approved as a part of the Prevention of Psychosis II (POP II) and Treatment and Intervention in Psychosis 2 (TIPS2) (Johannessen et al., 2001; Joa et al., 2015) projects by the Regional Ethical Committee (REK) of Western Norway (REK references: 84293 and 2011/1198, respectively). In addition to POP and TIPS inclusion and exclusion criteria, participants were excluded if they had a concurrent personality disorder or were suffering from an active psychotic or manic episode. All participants were either working at minimum part time or were students in high school or at a university.

Participants were identified as UHR using the Structured Interview for Psychosis-Risk Syndromes (SIPS) (Miller et al., 2003) upon inclusion in the POP 2 study. For inclusion in TIPS-2, the participant had to at least have suffered one psychotic episode and be diagnosed using the structured Interview for DSM (SCID) (Spitzer et al., 1992) or Kiddie SADS for individuals aged 13–17 (Kaufman et al., 1997). Weekly case meetings and discussions using case vignettes were used within the diagnostic team consisting of consulting psychiatrists and psychologists to reach diagnostic consensus.

### Interviews

First author HÅ conducted the interviews between 05.02.2021 and 03.04.2021. A semi-structured interview guide was developed from the recommendations made in Braun and Clarke (2023). Due to the inductive analysis format, questions were open-ended, allowing the participant to freely describe his pathway to care. The opening question was: “*Can you describe your pathway to care, from when you or someone else noticed that something was wrong, to when you received help in the mental health services?.”* A series of questions followed, pertaining to either internal factors such as the participants’ attitudes toward help seeking, or external factors such as how people in their surroundings affected their pathway to care. The interviews funneled from more open-ended questions to questions about the specifics of the experience as a means of letting the participant freely describe their experience whilst offering the possibility of clarifying any uncertainties or ambiguities in their stories.

All interviews were audiotaped (Mean duration: 34 m; Range: 20-44 m), with six interviews conducted over Norwegian Healthnet’s encrypted video-conference program, “Join” due to the Covid 19 pandemic, and three interviews conducted over telephone. All interviews were conducted-and transcribed verbatim in Norwegian.

### Analysis

We analyzed transcripts in ATLAS.ti 9, using thematic analysis as described in Braun and Clarke (2022) and used an inductive approach to develop a theoretical conceptualization of the pathway to care. The final interview was analyzed deductively post the development of the preliminary themes to test data adequacy, assessing whether any new or contradictory information emerged from this interview. Participants member-checked the results section post-analysis. They approved the findings, and no changes were made to themes or sub themes following member checking.

**Figure 1:**
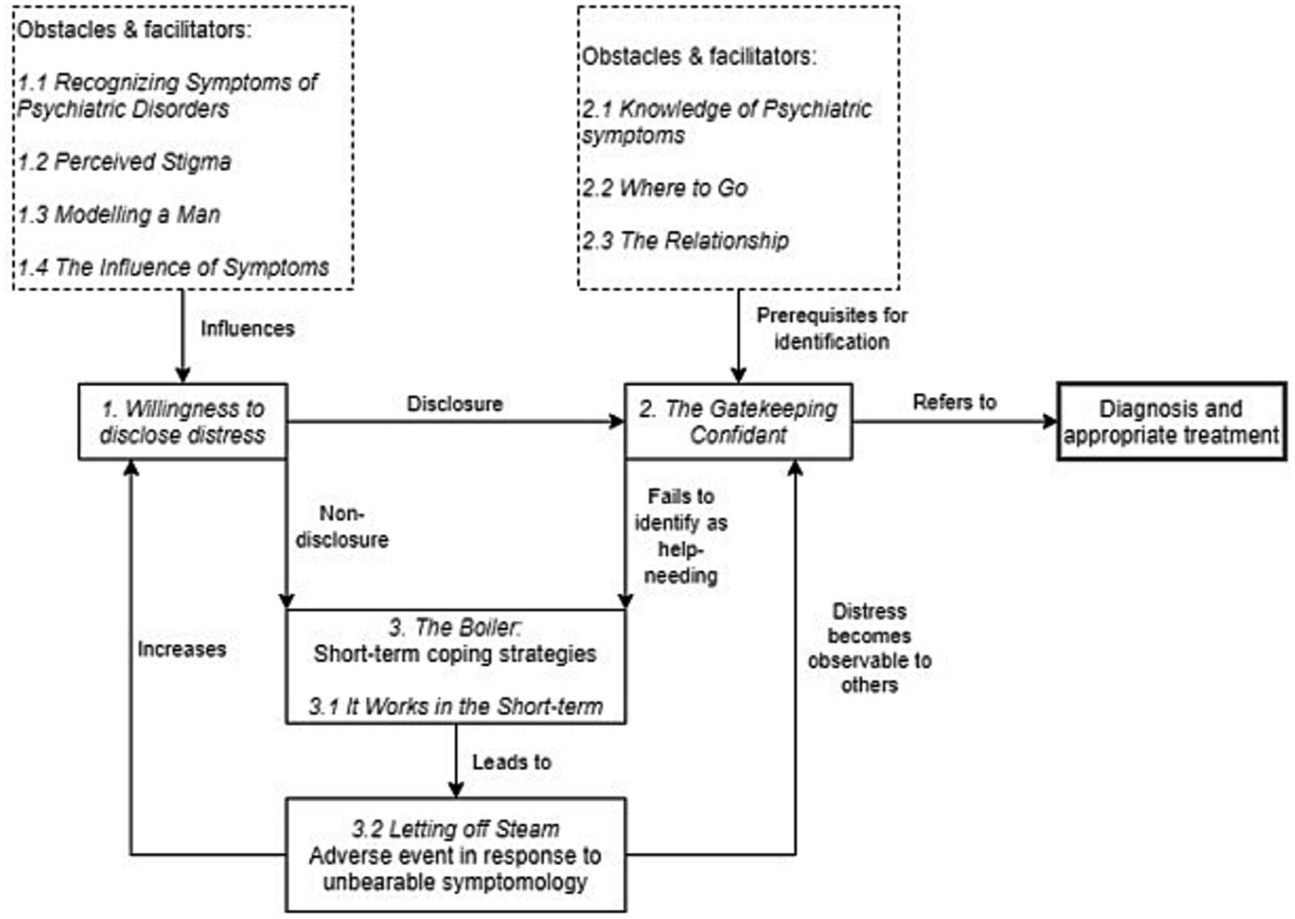
Conceptualizing the pathway to care.

HÅ conducted the analysis in collaboration with the other researchers/authors, using the following steps:

1. Thoroughly reading and re-reading all materials to become familiar with and gain a general impression of the interviews, as well as generating initial ideas about themes.
2. Generating codes through complete coding of the data set, including anything of interest. Each code could include more than one quote, and 502 codes were developed in this phase. Codes would reflect a semantic feature of the data set, such as quotes relating to the concrete behaviors of masking difficulties from family as “Hiding my difficulties from my family.”
3. Following coding, the codes were sorted into preliminary themes based on the similarity of their semantic content. Here, 63 candidate themes were developed, and the preliminary relations between themes were established.
4. Candidate themes and the relations between them were then refined through discussion with JC, JB, IJ, and WH, as well as through development of a thematic map, yielding nine sub themes and three core themes. These authors assessed the themes on their contribution to the research question, and on the consistency of codes within each theme, as well as whether each theme represented a unique aspect of the participants’ experiences, independent of other themes.
5. Authors then defined and named the themes, and established their position in the narrative through discussion. The conceptualization of the pathway to care (Figure 1) was consolidated, with sub-themes representing factors described by participants as affecting their pathway to care. Higher order themes reflect the underlying commonalities of corresponding sub themes.

### Researchers

The research team consisted of three clinical psychologists and senior researchers (JB, JC, IJ, and WH) and HÅ as a student in the clinical psychology program. All researchers had extensive experience working with individuals struggling with psychosis or high-risk states, as well as with research in the field. With the analysis mainly being conducted by HÅ, researcher preconceptions were disclosed in before conducting the study ensuring transparency regarding how prior assumptions, interests and biases may have influenced the analytic process.

## Results

A total of nine participants were interviewed, of which five men in the UHR group (Mean age = 19.5 years, SD = 1.66), and four in the FEP group (Mean age = 21.5 years, SD = 1.66). All participants in the UHR group were in employment, education, or training, compared to 75% in the FEP group. Eighty percent of the UHR and 100% of the FEP group consisted of men of Norwegian ethnic origin.

Analysis of the transcribed interviews yielded three core themes pertaining to obstacles and facilitators in pathways to care, consisting of nine sub-themes. The three core themes were named Willingness to disclose distress; Knowledge of psychiatric symptoms; and The Boiler. Together with these, the associated sub-themes were conceptualized as components and factors influencing a pathway to care (Figure 1). This pathway was conceptualized as a timeline beginning with the development of symptoms, and progressing until the participant was diagnosed with FEP or UHR and received appropriate treatment.

### Core theme: Willingness to disclose distress

All participants suffered from symptoms that did not manifest as observable signs for extended periods before becoming severe. Therefore, willingness to disclose – and being open about their distress-was described as important by all participants and seemed to be a prerequisite for being referred to-and receiving appropriate help in the mental health services.

#### Recognizing symptoms of mental illness

Being able to recognize changes in mental state as symptoms appeared to be a prerequisite for disclosing distress for several of the participants. Many noticed a change in their wellbeing, or started to experience attenuated symptoms, but failed to identify these as a part of potential mental illness. This appeared related both to the ability to recognize that something was wrong as well as to the ability to verbalize what they were feeling. For some participants, early symptoms were unspecific and difficult to distinguish from what they believed were normal fluctuations of mood and wellbeing. Some participants related the inability to recognize symptoms to a lack of knowledge of mental illness, and that they would have been more likely to disclose their distress at an earlier stage had they known that they were suffering from symptoms.

*“During that period, my mom would ask me all the time “is something wrong, are you sad, are you depressed?”, and so I believe I had become ill a lot earlier, that my course of illness was longer than I thought it was … Things had to become a lot worse [for me to be able to notice it].”* (“Kristian”)

#### Perceived stigma

Participants disclosed a variety of beliefs associated with mental illness or being a patient in mental health care. This appeared to influence their willingness to disclose distress. The most typical beliefs about mental illness were related to stigma and shame, negative beliefs about what others might think about them, and a perception of being alone in their suffering. The normalization of symptoms after disclosure and in treatment represented a sharp contrast to a feeling of being the only one struggling. Interviews indicated that it could lead to increased openness and acceptance as well as an increased willingness to disclose distress. All participants mentioned how normalization had helped them to become more open, suggesting that the stigma associated with mental illness was an important obstacle to seeking help:

*“I felt like there was no one else in the world who felt like I did, which meant that something was wrong with me. But my mom motivated me to seek help by telling me that thousands of people felt the same way, and that most people would suffer a depression at some point in their lives.”* (“Alexander”)

#### Modeling a man

All participants described in some way how masculine ideals had affected their willingness to seek help, directly or indirectly. A typical experience was that common beliefs about masculinity prevented them from disclosing distress, as men were supposed to be “strong” and “solid.” All nine interviews indicated that the men usually did not talk about their personal issues with friends, and that they would feel embarrassed to talk about what they were going through. However, an experience mentioned by several participants was that opening up to male friends modeled that openness for the rest of the group. For them. This appeared in turn to have resulted in more supporting friendships and an environment where they more easily could disclose their distress, as well as receive help and support more rapidly:

*“I could be open with those who knew me well. They learned to see it themselves, and were much quicker to ask about how I was doing … but with others I felt like I had to play the male role, but thinking about that, I think it’s completely wrong, It is completely normal to do, but I feel like it’s wrong because you can’t be yourself … I’ve been lucky, I realized that quite early.”* (“Fredrik”)

#### Sub theme: the influence of symptoms on disclosure

Symptoms associated with the UHR state and psychosis appeared to have affected the young men’s willingness to disclose distress through either increasing or decreasing it. Distressing symptoms had prompted some form of coping strategy in all participants. Disclosure was rarely the first choice, with participants rather resorting to social withdrawal and avoidance of situations. This could sometimes aggravate symptoms. However, aggravation of symptoms combined with unhelpful coping strategies appeared to make disclosure more likely. The participants disclosed varying degrees of awareness of their symptoms but were typically able to recognize that a change in their psychological wellbeing had occurred.

*“I started to see shadow-people … and spiders crawling on the walls, I was extremely paranoid, which made it really difficult to have a social life, so I isolated myself in my room to deal with it … it was even difficult to communicate with my sister who I lived with at the time.”* (“Svein”)

### Core theme: the gatekeeping confidant

*The Gatekeeping Confidant*-theme focuses on the confidants’ function as a gatekeeper. It appeared to be important for whether the participant was referred to the mental health services for assessment and treatment. The confidant could be anyone the participant confided in-or who in other ways noticed their distress; friends, family, teachers, general practitioners, or first-line mental health professionals. Some needed only to confide in a single person to be referred to mental health care. Others sought help with several persons, but who failed to recognize the need for professional help. The confidants had to correctly identify the participants’ distress as symptoms, and also be aware of the relevant sources of care such as the GP, TIPS, or other mental health care providers. Also, they would have to be able to motivate the participant to seek help, something that seemed to be influenced by the strength of their relationship.

*“We had a fairly good relationship, and so it was she who asked me whether I wanted to talk to someone professional. I didn’t really want to, but I did it for her […]. I had told her about my nightmares and the voice I was hearing, and it was because of this that she told me she didn’t have the competence to deal with it. She said I should talk to someone professional, and that’s when I got in touch with TIPS.”* (“Thomas”).

#### Knowledge of psychiatric symptoms

This sub-theme pertains to the ability of the confidant to recognize mental health symptoms in the participant when they were disclosed to or presented to them. Many participants experienced that other people took notice of strange behaviors or signs of their coping strategies, but few identified these as symptoms of mental illness. Despite “Martin” spending much time with his friends whilst psychotic, they were unable to detect his psychosis, and rather attributed it to his personality. Only when he started failing to function at work, he was referred to the mental health services by his manager, who had prior experience with seeing psychotic illness:

*“He kept an eye on me for a while, because he knew what he should be looking for. He had seen it himself a long time ago, in another person who worked there, so he had some experience with seeing people having a psychosis or something”* (“Martin”)

#### Where to go

Following a correct identification of symptoms of mental illness, the confidants acted as gatekeepers and influenced the pathway to care with their knowledge of where to refer the participant. The most common course of referral was through confiding in a trusted person, who then referred the participant to a general practitioner. He or she subsequently referred the patient to mental health services.

*“After I talked to my mom, she wanted me to talk to the general practitioner to hear what she thought and if she could help me. And after talking to her [The general practitioner] a couple of times I was referred to outpatient treatment at a regional psychiatric clinic (DPS)”* (“Alexander”).

#### The relationship

Participants would usually disclose to someone close, typically a parent, a best friend, or another person with whom they had a close relationship. Despite having disclosed their distress, participants were often reluctant to seek help. In these cases, a strong relationship with the confidant seemed to be a key motivator, as the confidant would convince the participant either that their symptoms required professional help, or the participant “*just did it to make them happy.”* Another aspect of the relationship was that the stronger and closer it was, the more likely the confidant would be to detect changes in affect and behavior.

*“My father saw it really quickly … We’re so similar, so he’s able to see the signs right away, so the second it started happening he knew it … […] I spent much time with him, and he has seen all my emotions, but this was new to him … and that he saw it, I think that was really important”* (“Fredrik”).

### Core theme: The boiler

Following disclosure of distress, two main trajectories were identified: Either the confidant would refer to appropriate health care, or he or she would not recognize the problems as symptoms requiring care and thereby obstruct access. Non-disclosure of distress also obstructed referral to mental health services. Both lead to an increase in distressing symptoms such as paranoia, racing thoughts, and hallucinations. With escalating symptomatic pressure and suffering, willingness to seek help seemed to increase, or adverse events in response to symptoms would occur, augmenting the probability of someone detecting the participant’s distress.

#### It works in the short-term

Regardless of what symptoms they suffered, several participants attempted to manage them merely by enduring, believing they were transient, or withdrawing socially. Social withdrawal was by some participants used either to relieve stress in social contexts whilst suffering symptoms, or as something developing over time without the participant having any rationale as to why. Despite the participants’ attempts to cope in solitude, their symptoms tended to develop in a way rendering these coping strategies insufficient in the long term. The strategies only served to mask their illness in the short term.

*“The voices could be quite loud, […] and sometimes I would hear this really manic laughter when I was talking to people […] It was easier to be alone than social because of the symptoms [Auditory hallucinations], so I started quite early to build a tolerance for being by myself”* (“Jonas”).

#### Sub theme: letting off steam

Participants described that following an increase in symptoms, suffering increased until they became unbearable, leading to the externalizing of distress. They described a variety of responses to insufferable levels of symptoms, such as suicidal ideation, or even in some cases responses such as violence toward others, suicide attempts, or experiencing extreme mood-swings.

*“I had written a letter and I was ready to just end it, but luckily I survived, and I think it just made me realize that I could get help, and so I chose that, I didn’t want to go through with it [Suicide]”* (“Jonas”)

## Discussion

This study has explored the complex interplay of factors influencing the pathway to care for young men at Ultra-High Risk for, or a first episode of, psychosis. The main findings appear to indicate a synergy between the young men’s symptoms, coping strategies, and agents assisting in the initiation of a pathway to care. Regardless of what coping strategies the participants used, a common issue appeared to be that they only were effective in the short term and led to progressively higher levels of distress. This in turn seemed to either motivate them to use disclosure as a coping strategy, or alternatively the levels of distress would become unbearable and culminate in an adverse event such as a suicide attempt or violent or bizarre behavior.

While commonly reported responses to symptoms appeared to be social withdrawal and attempts to endure symptoms, distress tended to present as external signs at worse levels of suffering. Social withdrawal can be a negative symptom but could also be a conscious and willed response to symptoms. Negative symptoms as a mix of core symptoms and coping strategies in our sample appeared to interact unfavorably with the environment in delaying help-seeking. The social withdrawal described by participants could thus be understood both as a symptom, as well as a coping strategy. Some participants described withdrawal as a means of avoiding aggravation of symptoms, whereas others described it as developing over time, without any explicit explanations. Regardless, social withdrawal might help understand the dynamics in help seeking for psychosis in men, as it appears to present as an obstacle to detection of symptoms and seeking help.

Among these participants, help-seeking was initiated by others in most of the nine cases, making disclosure of symptoms to a confidant a prerequisite for initiating a pathway to care. This tended to be delayed until an aggravation of symptoms led to the participant confiding in someone, or the severity of symptoms leading to behavior or situations making others able to detect their distress and them helping him. Better knowledge of mental illness in a confidant appeared to increase the likelihood of referral to mental health services.

The men in this study were typically reluctant to seek help. Despite being able to notice changes in their mental status, they did not perceive themselves as needing help until an aggravation of distressing symptoms. Obstacles to help-seeking such as masculine ideals, stigma, and inability to recognize early symptoms appeared to make them less likely to seek help, and to rather resort to endurance, which in turn failed to alleviate symptoms over time. This corresponds with previous qualitative research on perceptions of illness and pathways to care in both UHR and FEP. In Tanskanen et al. (2011) participants described suffering from symptoms of psychotic illness from weeks to years without realizing that they needed help. Further, in Bay et al. (2016) many participants held the belief that their symptoms were transient or a part of normal adolescent development. Hardy et al. (2009) describe a perception of need, and being influenced by the severity of symptoms such as paranoia, auditory hallucinations, and fluctuating mood as important prerequisites for seeking help. This is further supported in Falkenberg et al. (2015) where lower levels of distress in response to attenuated psychotic symptoms delayed help-seeking behavior.

Findings from other research indicating more negative attitudes toward mental health-seeking in males compared to females (Galdas et al., 2005; Wahto and Swift, 2016), and associating help-seeking with weakness and fear of social judgment, resonates with disclosures by several participants in our study. Indeed, the study by Wahto and Swift (2016) showed that social stigma, self-stigma and gender role conflict can explain much of the variance in men’s negative attitudes.

Findings regarding willingness to disclose distress can also be understood in the context of masculine ideals, and perceptions of the male gender role (Wahto and Swift, 2016). For instance, in research on pathways to suicidal action in men, non-disclosure was found to be a common behavior hindering detection by others of distress (Cleary, 2005). The men in this study described disclosure of distress as implying weakness, and consequently projected a façade of strength to their surroundings, hindering others from detecting their distress. This is similar to the findings from our study, where help-seeking and disclosure of distress by several were perceived as signs of weakness. On the other hand, critique has been raised regarding current operationalizations of masculinity. Previous quantitative research has been biased toward viewing masculinity as a stable construct, as opposed to qualitative research which has tended toward conceptualizing it as more fluid and multi-faceted, for example including features that may be beneficial for overcoming depression. Some researchers suggest future research to include positive dimensions of masculinity, such as male relational styles and courage, as a way to counteract the common conceptualization of masculinity which the authors deem reductionistic and simplistic (Seidler et al., 2016). Nevertheless, stereotypical ideas of masculinity appeared to delay help-seeking in the participants in our study.

To whom the participant in this study disclosed their distress, or who in other ways were able to detect their distress, seemed to play a key role in the participants’ pathway to care, either facilitating or obstructing the pathway. This is in line with other research on pathways to care for general mental health complaints, where the individual (men and women) would tend to initiate help-seeking independently only in around 16% of cases (Mac Donald et al., 2018). There appears to be no reason to believe this would be any different in men-only groups. Further, family, friends, and other immediate relations are often the first to detect changes in a person suffering from psychosis, often in response to an exacerbation of symptoms, suicidal ideation or aggression toward the self or others (Judge et al., 2008). However, in a psychosis risk stage, individuals commonly cope themselves. Combined with social withdrawal, symptoms may then develop undetected and eventually lead to an adverse event, eliciting either a need to disclose or making the need for help clear to others. Avoidant coping strategies such as social withdrawal are associated with a higher degree of psychopathology, and delays in detection of distress in males (Livingston et al., 2021). Connor et al. (2016) reported similar findings regarding the role of the family system in help seeking for FEP. They found that emotional and social withdrawal was the main obstacle to family noticing psychotic symptoms. In some participants in this study bizarre behavior was interpreted as a “teenage phase.” Thus, the participants’ coping strategies and symptoms could be hypothesized to have interacted with the ability of others to detect symptoms in a way that delayed help-seeking.

Spreading awareness and non-stigmatizing easy access to mental health care appear to remain important to shorten duration of untreated psychosis and psychosis risk, however more focus on social withdrawal and “quiet” symptoms may need more room in awareness campaigns.

### Strengths and limitations

A limitation regarding the sample was that the men who were identified at UHR had sought and received help at some point. Thus, the group of UHR men who do not seek help before either remitting or transitioning into psychosis have not been interviewed. This may in some ways have been controlled for by interviewing those who had transitioned into FEP as they per definition had not sought help before transitioning, consequently illustrating a pathway to care in which one does not seek help in the prodromal phase.

An aspect regarding the generalizability of the findings is the context in which the participants are situated. The participants were all part of a catchment area with a strongly developed system for detection of-and intervention with psychosis, thus possibly making the findings less likely to occur in other catchment areas. Health system variables may be assumed to be less likely to disrupt and hinder help-seeking here compared to other places without such early detection efforts.

Qualitative representativeness pertains to selecting participants who can provide the most representative information to the research question, and consequently, including both UHR and FEP men could have contributed to increased representativeness. The pathways to care for these groups were different, with the FEP participants having a pathway in which they were not detected until transitioning into psychosis. This heterogeneity of experiences allowed for inquiry into a wide spectrum of factors influencing pathways to care for the young men. On the other hand, the group was homogenous with regards to representativeness of ethnic minorities with only one participant being non-white.

The prominent role of HÅ, particularly related to conducting the interviews in this study might have made research process more susceptible to bias of the researcher, allowing for the possibility of both interviews and analysis to be skewed by bias. Attempts were made to control for this by disclosing expectations and possible bias before initiating the research process, designing an interview guide restricting the interviews to the structure of the guide, as well as discussing findings with other researchers. Member checking was used to exclude the influence of bias by allowing the participants to validate our findings. Despite this, one can never rule out that implicit biases may have affected the results. This may on the other hand be contrasted with other research employing both qualitative and quantitative methods, where findings seem to be similar, increasing the validity of our findings.

### Implications

With the current paradigm of psychosis research focusing on preventive interventions, effective detection of at-risk individuals remains the key to intervention in the prodromal stage of illness (Fusar-Poli et al., 2020). A preventive approach to altering the course of psychosis poses questions regarding the optimal strategy for identifying at-risk individuals, as well as assessing who should be treated (Millian et al., 2016). This study provides insight into a sub-group of the high-risk population notoriously difficult to detect.

Findings in our study indicate a problematic interplay between symptoms, stigma, and participants’ as well as people in their immediate relations’ ability to recognize early signs of potentially severe mental illness. Like findings by Tanskanen et al. (2011), difficulties in detecting the early symptoms of illness seem to increase the risk of a pathway to care involving crisis or transition into a first-episode psychosis in the UHR population. With people in immediate relations of the participants seemingly being the primus motor for instigating help-seeking, inquiry into how to make the public more able to recognize early signs should be relevant. Accessibility of services is also crucial; outreaching low threshold early detection teams have also proven effective in reaching young men compared to service entry as usual (Johannessen et al., 2007).

Participants commonly presented with unspecific signs of illness in the prodromal stage, if they disclosed any signs at all. However, the most common sign in this study of a beginning mental illness appeared to be social withdrawal. Passive social withdrawal has been found to primarily function as a negative symptom, whereas active social withdrawal seems to be more related to positive symptoms (Hansen et al., 2009). Social withdrawal as part of negative symptoms such as avolition and reduced affect is associated with a worse functional outcome (Milev et al., 2005; Fusar-Poli et al., 2020). Our group recently found that “reduced expression of emotion,” a psychosis risk negative symptom, was the one symptom most predictive of transition to psychosis (Bjornestad et al., 2022a). There is little doubt that negative symptoms may be important in a trajectory toward psychosis. They are difficult to recognize and appear to become observable to others only when associated with a change in functioning and behavior, first and foremost as social withdrawal. However, even then they may be confused with phenomena such as pubertal lethargy, heartbreak, substance use, or general “teenage misery.” Thus, more knowledge and public awareness of negative symptoms associated with psychosis and psychosis risk could help increase the ability of key agents to instigate help seeking.

Knowledge and awareness pertain to mental health literacy. In the Stavanger catchment area, the average duration of untreated psychosis was reduced from 16 to 5 weeks (Melle et al., 2004) through a combination of information campaigns targeting mental health literacy and early detection teams. The lay perception of psychotic disorders however seems to be oriented toward positive symptoms (Jorm et al., 2005), and in the study by Connor et al. (2016), help-seeking was commonly delayed by persons in the immediate relations of the individual not being able to recognize negative symptoms as part of a potential psychotic prodrome. Including negative symptoms in mental health literacy campaigns, combined with easy access, could provide the individual and significant others with the tools to seek help at an earlier stage (Gronholm et al., 2017).

Finally, to counteract the negative effects of male role stereotypies, enriching psychosis prevention programs with interventions focusing on men could be relevant for inquiry. In a meta synthesis by Sagar-Ouriaghli et al. (2019), several approaches to improving male help-seeking for mental disorders are presented. Normalizing symptoms and reducing stigma, using more solution focused frameworks for help, and addressing male stereotypies of strength and responsibility are all strategies suggested in the study.

## Conclusion and future directions

In this qualitative study, we have explored factors influencing pathways to care for young men at risk of developing psychotic illness. Willingness to disclose distress and the ability of others to detect the development of illness appeared to be key factors facilitating an easy pathway to care. Thus, help-seeking in the young men was delayed primarily because they were reluctant to disclose their distress or because significant others were unable to accurately recognize symptoms. More research and inquiry into the effects of masculine stereotypes on help-seeking might be warranted to increase help-seeking. However, it may appear that information, awareness and easy access to care remain important in early detection and intervention in psychosis and psychosis risk, but that more emphasis should be placed on de-stigmatizing mental health problems in men.

## Data availability statement

The datasets presented in this article are not readily available because no data except interview transcripts were used for this study. These have been deleted after analysis. Requests or questions regarding data sets should be directed to wenche.ten.velden.hegelstad@sus.no.

## Ethics statement

The studies involving humans were approved by Regional Ethical Commitee of Western Norway reference numbers 84,293 and 2011/1198. The studies were conducted in accordance with the local legislation and institutional requirements. The participants provided their written informed consent to participate in this study.

## Author contributions

HÅ: Conceptualization, Data curation, Formal analysis, Investigation, Methodology, Writing – original draft, Writing – review & editing. JC: Conceptualization, Data curation, Formal analysis, Methodology, Resources, Supervision, Writing – review & editing. JB: Conceptualization, Data curation, Formal analysis, Investigation, Methodology, Writing – review & editing. IJ: Conceptualization, Data curation, Investigation, Methodology, Project administration, Writing – review & editing. WH: Conceptualization, Data curation, Formal analysis, Funding acquisition, Investigation, Methodology, Project administration, Resources, Writing – original draft, Writing – review & editing.
